# Bullying among medical students in a Saudi medical school

**DOI:** 10.1186/1756-0500-5-335

**Published:** 2012-07-02

**Authors:** Hasan Ali Alzahrani

**Affiliations:** 1 Department of Surgery, Faculty of Medicine, King Abdulaziz University, Jeddah, Saudi Arabia

**Keywords:** Medical, Students, Bullying, Harassment, Mistreatment, Educational environment

## Abstract

**Background:**

Bullying and sexual harassment of medical students by their teachers appears to be widespread phenomenon. However, nothing is published about its prevalence in conservative countries such as Saudi Arabia. This survey aims to ascertain the extent of these mistreatments among students in a Saudi medical school.

**Findings:**

A cross-sectional questionnaire survey was conducted on a group of 542 clinical years’ medical students in a Saudi medical school to explore students' perceptions of their educational environment including exposure to different kinds of bullying. Bullying was defined as “a “persistent behaviour against a medical student that is intimidating, degrading, offensive or malicious and undermines the confidence and self- esteem of the recipient”. Results revealed that more than one quarter (28.0%) of the surveyed students reported exposure to some sort of bullying during their clinical. Ninety percent of the reported insults were verbal, 6% sexual and 4% physical. Males were more exposed but difference was not statistically significant.

**Conclusions:**

Bullying among Saudi medical students is an existing problem. A policy against bullying and harassment should be adopted in all of medical colleges to monitor this phenomenon and support students who have been bullied.

## Findings

Bullying, harassment and intimidation of medical students can poison the educational environment. Bullying and sexual harassment of medical students by their teachers appears to be widespread globally. Several studies from various countries have shown that bullying, harassment, abuse or belittlements are a regular phenomenon faced by medical students [1-5]. No medical school is immune, irrespective of geographical location or cultural background [1,2]. In Pakistan [1], Ahmer et al. conducted a cross- sectional questionnaire survey on bullying of final year medical students in six medical colleges and found that 52% of respondents reported that they had faced bullying or harassment during their medical education, about 28% of them experiencing it once a month or even more frequently. The overwhelming form of bullying had been verbal abuse (57%), and consultants were the most frequent (46%) perpetrators [1]. In a Jordanian study, 61% of the students had experienced at least one form of mistreatment; as (52%) were exposed to verbal (shouting and humiliation); (32%) physical harm and (33%) sexual harassment [2]. However nothing is known about its prevalence among medical students in strictly conservative countries such as Saudi Arabia SA. Based on the socio-cultural characteristics of SA, there is a general impression that bullying is not a significant problem particularly the sexual harassment in SA. This survey aims to ascertain the extent of the various forms of mistreatments among medical students in a Saudi medical school.

## Methods

A cross-sectional questionnaire survey was conducted in Jeddah, SA on a group of 542 clinical years’ medical students of King Abdulaziz medical school to explore students' perceptions of their educational environment including exposure to different kinds of bullying. The targeted population was medical students who had completed at least one clinical year [n = 1196 students]. There was a slight preponderance of males (54.6%) over females (45.4%) in different grades, but this difference is not statistically significant (*p* > 0.05). They represent approximately 50% of the total number of students in the medical college. All students were invited to answer the survey questions about exposure to any form of bullying. The response rate was 45% (n = 542). The students were sub-grouped randomly by the college vice-deanship at the beginning of every academic year. Bullying was defined as “a “persistent behaviour against a medical student that is intimidating, degrading, offensive or malicious and undermines the confidence and self- esteem of the recipient”. Forms of bullying included verbal mistreatments such as belittlement, sexual harassment and physical abuse. All medical students were asked to answer a direct question on history of exposure to any form of bullying caused by a medical teacher in the previous academic year. Medical teachers were defined as clinical tutors and academic staff. Other categories such as senior students or peers were excluded as females were separated from males during clinical sessions.

The study was approved by King Abdulaziz University Hospital Bioethical Committee and the Dean of Faculty of Medicine, KAU, Jeddah, Saudi Arabia. Data analysis was performed using the Statistical Package for Social Sciences (SPSS) version 16. Data were presented using descriptive statistics in the form of frequencies and percentages for qualitative variables. Qualitative variables were compared using the Chi-square test. Whenever the expected values in one or more of the cells in a 2x2 table was less than 5, the Fisher exact test was used instead. Statistical significance was considered at *p*-value <0.05.

## Results and discussion

More than one quarter (28.0%) of the surveyed students reported exposure to some sort of bullying during their clinical rotations. Ninety percent of the reported insults were verbal abuse and belittlement, 6% sexual harassment and 4% physical abuse. The percentage increased significantly with maturation of students from 5^th^ year students (18.5%) through 6^th^ year students (35.5%) till it reached its peak at the beginning of the internship year with 43.7% (*p* < 0.05), Table 1. Males were more exposed than females but difference was not statistically significant. However, the percentage of females who had been exposed to sexual harassment was higher than males (9.8% vs 3.4%). The sexual harassment and physical abuse incidents were not reported legally by students for various reasons. The current survey of prevalence of bullying among medical students was part of a larger educational environment survey to assess students’ perception of their educational environment. Bullying, harassment and intimidation of medical students by medical teachers can poison the educational environment. Many reports of this were published worldwide in the last decade [1-5]. Comparison between bullying rates in various published studies is difficult due to the different socio-cultural characteristics of the studied populations and different methodologies used by researchers. However, no medical school is immune, irrespective of geographical location or cultural background.

**Table 1:** Exposure of students to bullying

**Insult**		**Years**			**Total**	p*
		**5**^**th **^**year**	**6**^**th **^**year**	**Intern**		
Exposure to bullying	Yes	50(18.5%)	71(35.5%)	31(43.7%)	40(56.3%)	0.000
	No	221(81.5%)	129(64.5%)	40(56.3%)	390(72.0%)	
Typeof bullying	Verbal	43(86.0%)	61(88.4%)	31(100.0%)	135(90.0%)	
	Physical	4(8.0%)	2(2.9%)	6(4.0%)	NA	
	Sexual	3(6.0%)	6(8.7%)	------------	9(6.0%)	

6. * Based on Chi Square.

This is the first published report from SA. The overall rate of bullying in the current study is 28% which is much lower than rates reported in developed countries. In USA, 42% of medical students reported harassment and 84% belittlement during their stay in medical school compared to 50% in Finland [3,4]. It is also lower than some of Middle East countries’ rates such as Jordan 61% and Pakistan 52% [1,2]. The percentage of those exposed steadily increased as students went through the three clinical levels. Similar to most of previous studies, the commonest form of bullying in the current study was the verbal abuse which includes shouting, humiliation and belittlement. Sexual harassment’s rate is relatively much lower in our study compared to prevalence rates reported in industrialized countries (18-60%) [5]. One in 3–5 Dutch female medical students had experienced unwelcome sexual attention from patients, colleagues or supervisors [5]. The current rate is even lower than rate reported in Jordan which shares some common cultural backgrounds with SA (6% vs 33%) [2]. Only 6 females and 3 males were exposed to such form of abuse and did not report the incidents. The discussion of bullying and particularly sexual harassment is sensitive and embarrassing in local culture. This may lead to underreporting of the cases. This study was limited to bullying caused by consultants, clinical professors and tutors as they were reported as the frequent perpetrators in previous studies [1-3]. Future national study should take the inherent weaknesses and limitations in consideration. This may include a more detailed questionnaire which defines all forms of bullying in more details particularly the sexual one. It may be better to display it on a designated website as that may ensure confidentiality of the respondents’ identity and that will encourage a higher response rate in females.

In conclusion, bullying rates in this study are relatively lower than similar rates in literature. This may be attributed to under-reporting and cultural factors. A policy against bullying and harassment should be adopted in all of medical colleges to monitor this phenomenon. Bullying rates may be reduced through staff development and establishing a system for monitoring these abuses to medical students. There is also need for active effective student support units. A wider national study is warranted to explore this phenomenon further in SA and countries of similar cultural backgrounds.

## Competing interests

The author declares that he has no competing interests.

## Authors’ contributions

The corresponding author (HA) contributed to concept, design, supervision of study’s conduct, data collection, writing of the manuscript and submission. The author read and approved the final manuscript.
